# African science contributes to the fight against cancer in the world: reflections after the 10th AORTIC conference, 18–22nd November 2015, Marrakesh, Morocco

**DOI:** 10.3332/ecancer.2016.ed52

**Published:** 2016-02-01

**Authors:** DC Stefan

**Affiliations:** President elect AORTIC, South African Medical Research Council, Parrow, Cape Town 7550, South Africa

**Keywords:** Africa, cancer, oncology, science, research, Aortic

## Abstract

AORTIC (OAREC), the Pan – African organization aiming to unite African scientists in the fight against cancer on the continent, held its 2015 conference in November, in Marrakesh. It was an opportunity to evaluate the progress made, to strengthen scientific collaborations and to draw the future battle lines against cancer on the continent.

## Cancer in Africa

As I take time to reflect after the last conference organized by AORTIC in Marrakesh, in November 2015, a question comes to my mind: “Can Africa in the 21st century make the substantial contribution to science that is so much needed?” I still need to find a response.

In a world dominated by health inequalities, war and refugees, climate change and malnutrition, Africa remains a continent characterized by chronic socio economic problems and political instability with minimal or insignificant investment in cancer research and education.

Africa has one of the youngest populations, with almost half of its inhabitants younger than 15 years of age [1], and it is a continent where the life expectancy is low [2] but increasing steadily. It is a continent of opportunities for growth, undergoing massive economic and social transformations. Africa is acknowledged to be the continent where great humanitarians have been born and led by example, among them Nelson Mandela and Desmond Tutu, a continent which has served as a model to all and where US presidents and billionaires have made their way to come and be inspired.

Estimates suggest that the annual number of new cancer cases here will grow over the next five years to above 1 million [3], but the supporting data are sparse. We have more than a 1 billion people living in our continent but, according to the World Bank, almost half (42.7%) of those living in sub-Saharan Africa earn less than US$1·90 per day [4]; the continent speaks more than 2000 different languages, but some of them don’t even include a word for cancer.

In November 2015, AORTIC (African Organization for Research and Training in Cancer) held its Conference in Marrakesh, Morocco, with the title: “AORTIC Roadmap to Cancer Control in Africa”. It was attended by almost 1000 participants.

AORTIC [5] is an African based non-governmental organization, dedicated to the promotion of cancer control and palliation in Africa. The organization strives to unite the African continent in achieving its goal of a cancer-free Africa, and seeks to make a positive impact throughout the region through collaboration with health ministries and global cancer organizations. Formed in 1983 by expatriate African cancer care workers, scientists and their friends, the organization was dedicated to the promotion of cancer control in Africa. After a pause of a few years in its activities, it restarted its mission in 2003 led by presidents from Tanzania, Senegal, Cameroon, Nigeria and Egypt.

The key objectives of AORTIC are to further the research relating to cancers prevalent in Africa, support the management of training programs in oncology for health care workers, deal with the challenges of creating cancer control and prevention programs and raise public awareness of cancer in Africa.

The Marrakesh meeting brought together scientists from more than 42 countries around the world in more than 112 sessions, including the pre-conference sessions. The conference included early morning roundtables, keynote lectures, plenary sessions, and poster and oral presentations on a large range of subjects. Exciting topics included in the program were discussions on establishing cancer research priorities in Africa, a symposium on BRICS, the impact of inequity on cancer care, health economics and national cancer control plans.

The African Cancer Leadership Institute continued its activity with interactive sessions and a special evening was organized by the newly launched Pan-African Women’s Association of Surgeons (PAWAS) and the Office of Women Faculty Programs of the MD Anderson Cancer Center.

Cancer registration in Africa set the tone as a preconference workshop, with a full session organized by the African Cancer Registry Network, the hub for registry in Africa. Breast cancer and cervical cancers remained the focus of many presentations in the category of female cancers and together with haematological malignancies, pathology and HIV related cancers attracted a large audience.

As almost a third of cancers in Africa are caused by infections, prevention thereof remains pivotal on the continent, so a special emphasis was placed on vaccination and preventive measures.

A lot of discussions and active engagement took place in the sessions addressing prostate, lung and colorectal cancer, with African scientists leading the international forum.

Innovation, telemedicine and the role of twinning (the program’s African-Italian cooperative oncology group (AFRICOG experience)) were also included in the program.

Remarkable international organizations such as The National Cancer Institute, The International Agency for Research on Cancer, The American Cancer Society, The World Health Organization, The American Society for Clinical Oncology, The International Union for Cancer Control, The American Association for Cancer Research, and many others contributed to the success of the conference.

Besides these highlights, there were many other workshops and sessions which took place during the conference and for the two preceding days. The topics ranged from cancer risk assessment, genetic counselling, curable cervical cancer in Africa, the science of global cancer disparities, the impact of e-learning methodology in African settings, to disease control priorities in developing countries, biobanks, resource mobilization for cancer control and further to psycho-oncology, malignancies in the setting of HIV infection, priority actions for comprehensive cancer control interventions in African countries, and sexuality and cancer.

As I reflect back to a conference which not only provided a diversity of topics and themes for debate, but also showcased research results and progress in science on the continent, great ideas and original views, a conference which left me richly empowered by networking with old and new friends, the same question comes back to my mind: “Can Africa in the 21st century make the substantial contribution to science which is so much needed?”

I now have the answer, without any doubt.
